# First in human surgical implantation of a leadless pacemaker on the epicardial portion of the right atrial appendage in a patient with a cardiac electronic devices mediated dermatitis

**DOI:** 10.1093/icvts/ivac050

**Published:** 2022-02-28

**Authors:** Ivan Eltsov, Antonio Sorgente, Carlo de Asmundis, Mark La Meir

**Affiliations:** 1 Cardiac Surgery Department, Universitair Ziekenhuis Brussel—Vrije Universiteit Brussel, Brussels, Belgium; 2 Heart Rhythm Management Centre, Postgraduate Program in Cardiac Electrophysiology and Pacing, Universitair Ziekenhuis Brussel—Vrije Universiteit Brussel, European Reference Networks Guard-Heart, Brussels, Belgium

**Keywords:** Leadless pacemaker, Epicardial pacing, Pocket complication, Sick sinus syndrome

## Abstract

A 33-year-old woman with Sick Sinus Node syndrome and persistent atrial fibrillation underwent a Maze IV procedure in order treat atrial fibrillation and concomitant atrial epicardial implantation of a leadless pacemaker to manage her sinus node insufficiency. Last option has been chosen due to rare pocket complication after previous classic dual-chamber pacemaker implantation.

## INTRODUCTION

Sick sinus syndrome is the disease caused by intrinsic abnormal impulse formation and/or propagation from the sinus node. This condition often requires pacemaker implantation with the preference of atrial or dual-chamber pacing in order to prevent and treat atrial tachyarrhythmias driven by ventricular pacing [1]. One of the main contraindications for classic pacemaker implantation is pocket complications and the only alternative is leadless pacemakers. However, leadless pacemakers have limitations as well and their on-label use is the transvenous implantation in ventricle position only. We report the first in human, to our knowledge, case of epicardial implantation of leadless pacemaker in the atrial position during concomitant cardiac surgery.

## CASE REPORT

**Figure 1:**
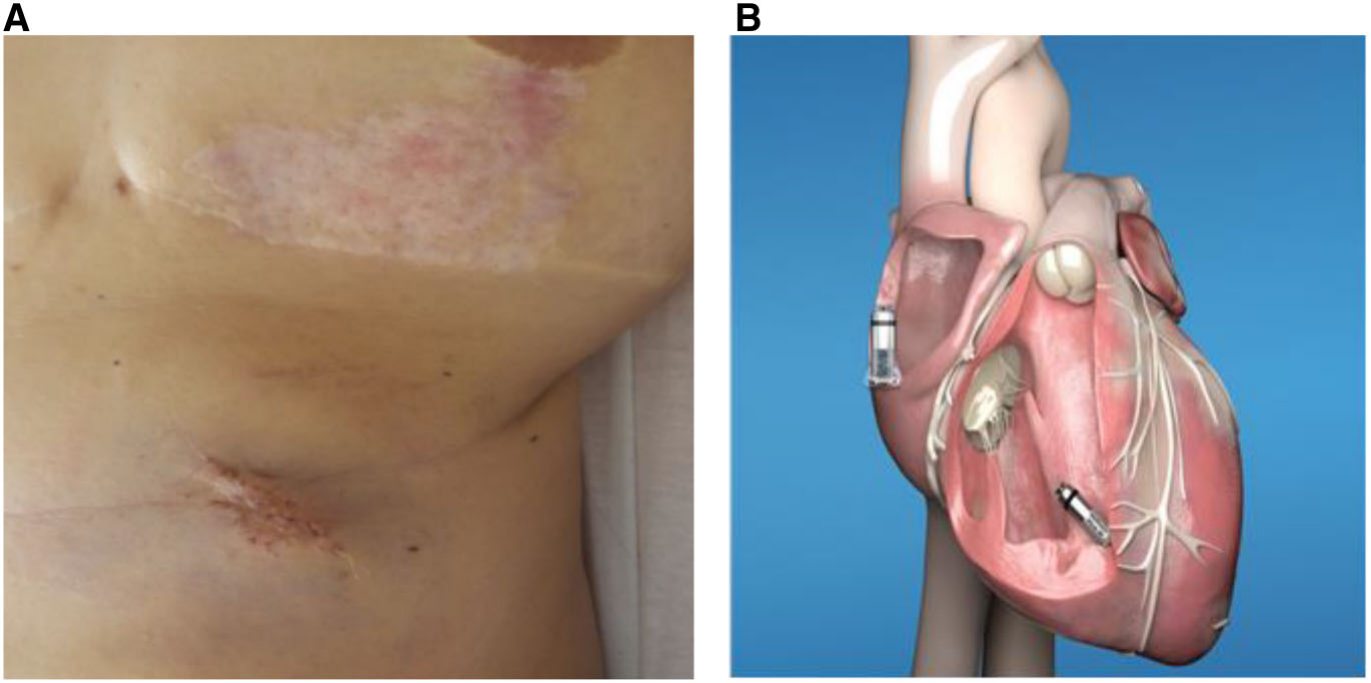
(**A**) Pyoderma gangrenosum on the site of explanted classic pacemaker. (**B**) Schematic of epicardial positioning of 2nd Micra pacemaker.

A 33-year-old female patient, with a history of drug refractory inappropriate sinus nodal tachycardia and a consequent hybrid inappropriate sinus nodal tachycardia ablation procedure, underwent implantation of a subcutaneous Reveal Linq device, in order to control the heart rate in the post-procedural period. One month after implantation, the device was explanted due to the development of a device mediated dermatitis. Patient did well for the following 2 years, after which she developed sick sinus syndrome and significant chronotropic incompetence. A dual-chamber pacemaker capable of rate responsiveness was implanted in the left subcostal region. One year after the implantation, the device and its leads were explanted due to the recurrence of skin complication at the level of the pacemaker pocket (Fig. 1A). A pyoderma gangrenosum was then diagnosed. This is a rare neutrophilic dermatosis characterized by painful, necrotic ulceration, diagnosed after exclusion of infection and allergy driven dermatitis [2]. Given the intolerance to all subcutaneous devices and the need of a higher heart rate, a leadless pacemaker (Micra AV, Medtronic, Minneapolis, MN, USA), able to provide AV synchronous (VDD) pacing was implanted in the apical septum of the right ventricle. A few months after implantation of the leadless pacemaker, the patient unfortunately developed paroxysmal atrial fibrillation, which impaired significantly the synchronization between atrial spontaneous depolarizations and ventricular pacing. After a multidisciplinary team discussion, a decision was made to perform a surgical atrial fibrillation treatment (Maze IV) with concomitant implantation of a second leadless pacemaker in the epicardial portion of the right atrial appendage (Fig. 1B). A pacemaker with epicardial leads was not considered a feasible option, due the previous history of devices’ related pyoderma gangrenosum. Decision has been discussed with local ethics committee and patient has signed informed consent form.

**Figure 2:**
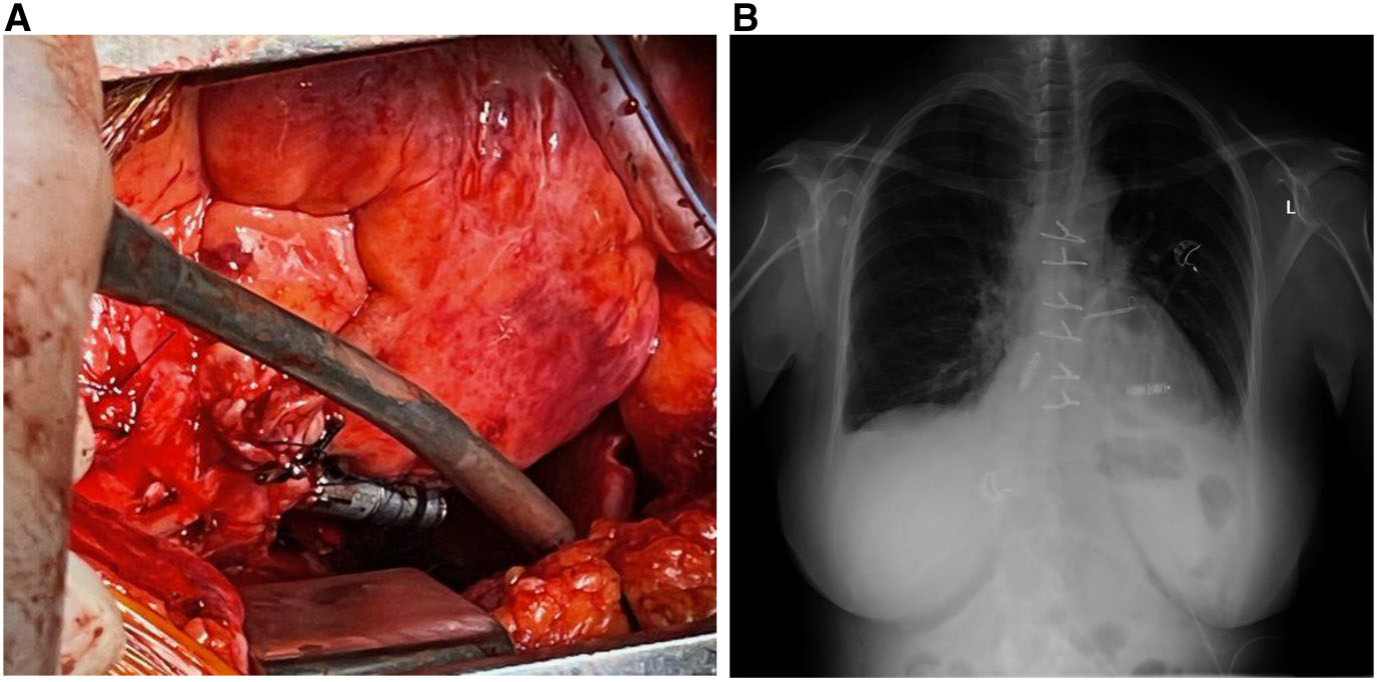
(**A**) Intraoperative photo of epicardial leadless pacemaker implantation. (**B**) Chest X-ray during follow-up visit.

Maze procedure was performed using median sternotomy, which allowed a direct visualization of the right ventricle. Pacemaker implantation was performed after removal of the aortic clamp and stopping bypass but with cannulas kept in place as a security measure. Micra device was ejected from the delivery system, given the fact that the original fixation mechanism was not meant to be used. Micra leadless pacemaker was fixed to the epicardial portion of the right appendage by means of 2 sutures (Ethibond 2-0): its cathode was stitched to right atrial appendage and its anode to the right atrial free wall (Fig. 2A). The implanted device showed satisfactory electrical parameters (Table 1). After removal of both intracardiac cannulas, pericardium was almost entirely sutured. The intervention was brought to the end in the usual fashion (decannulation, haemostasis check, thorax drain and final closure). Before final suturing of the sternum, device was interrogated again and showed still good electrical parameters (even improved due to partial closure of pericardium; Table 1).

**Table 1:** Device parameters after implantation and 3 months of follow-up

	Implantation	Three months of follow-up
Pace mode	VVIR (AAIR) 60	VVIR (AAIR) 60
V pacing (A pacing)	N/A	45%
Sensing	3.8 mV	4.0 mV
Threshold	0.5 V@0.24 ms	0.5 V@0.24 ms
Impedance	710	725

The Micra Device implanted in the right atrial epicardium was programmed at VVIR (AAIR) 60 mode, whereas the previously implanted Micra leadless pacemaker located in the right ventricle was reprogrammed to VVI 40 (ventricle sensing/pacing, inhibited while hazard ratio is over 40), as a backup option with disabled atrial detection function.

At the classical 1 and 3 months of follow-up, no signs of devices’ dislodgment were noted on the chest X-ray (see Fig. 2B) and no significant electrical parameter change was recorded—see Table 1.

## DISCUSSION

Severe pocket complications are rare entity and these patients are good candidates for percutaneous leadless pacemaker implantation after explanting of classic device [3]; however, existing leadless devices implantation method is limited by the fact that these devices are set up to pace only the ventricles. It has been clearly shown in the scientific literature that this modality of pacing is not optimal for patients with normal atrioventricular nodal function because it causes asynchrony between atria and ventricles. This lack of coordination between atrial and ventricular contraction can favour a left ventricular systolic dysfunction and the development of pacemaker syndrome [4]. Earlier in 2020, Backhoff *et al.* [5] have performed an animal study on epicardial implantation of a leadless pacemaker on both atria and ventricles, showing good electrical performance and no macroscopic dislodgment after 31 weeks of follow-up. Whether the rationale for standalone surgical implantation of a leadless pacemaker remains debatable due to invasive character of this technique compared to other therapy alternatives, concomitant leadless pacemaker implantation on patients already scheduled for cardiac surgery including sternotomy is relatively simple from the technical standpoint and does not increase periprocedural complications, ensuring correct bradyarrhythmia therapy with no risk of pocket or lead complications.

**Conflict of interest:** Carlo de Asmundis receives research grants on behalf of the centre from Biotronik, Medtronic, Abbott, LivaNova, Boston Scientific, AtriCure, Philips and Acutus; Carlo de Asmundis received compensation for teaching purposes and proctoring from Medtronic, Abbott, Biotronik, Livanova, Boston Scientific, Atricure and Acutus Medical Daiichi Sankyo. Mark La Meir is consultant for Atricure. Ivan Eltsov receives compensation fees from Medtronic.

## Reviewer information

Interactive CardioVascular and Thoracic Surgery thanks the anonymous reviewers for their contribution to the peer review process of this article.
